# Supplementary material for: Bodo saltans (Kinetoplastida) is dependent on a novel Paracaedibacter-like endosymbiont that possesses multiple putative toxin-antitoxin systems
Source: ISME J. 2021 Jan 15;15(6):1680–94. doi: 10.1038/s41396-020-00879-6 (PMC8163844; doi:10.1038/s41396-020-00879-6)
Supplement: Supplementary file 1 — Supplementary Figures 1-8 [file 41396_2020_879_MOESM1_ESM.docx]

**Supplementary Data**

*Bodo saltans* (Kinetoplastida) is dependent on a novel *Paracaedibacter*-like endosymbiont that possesses multiple putative toxin-antitoxin systems


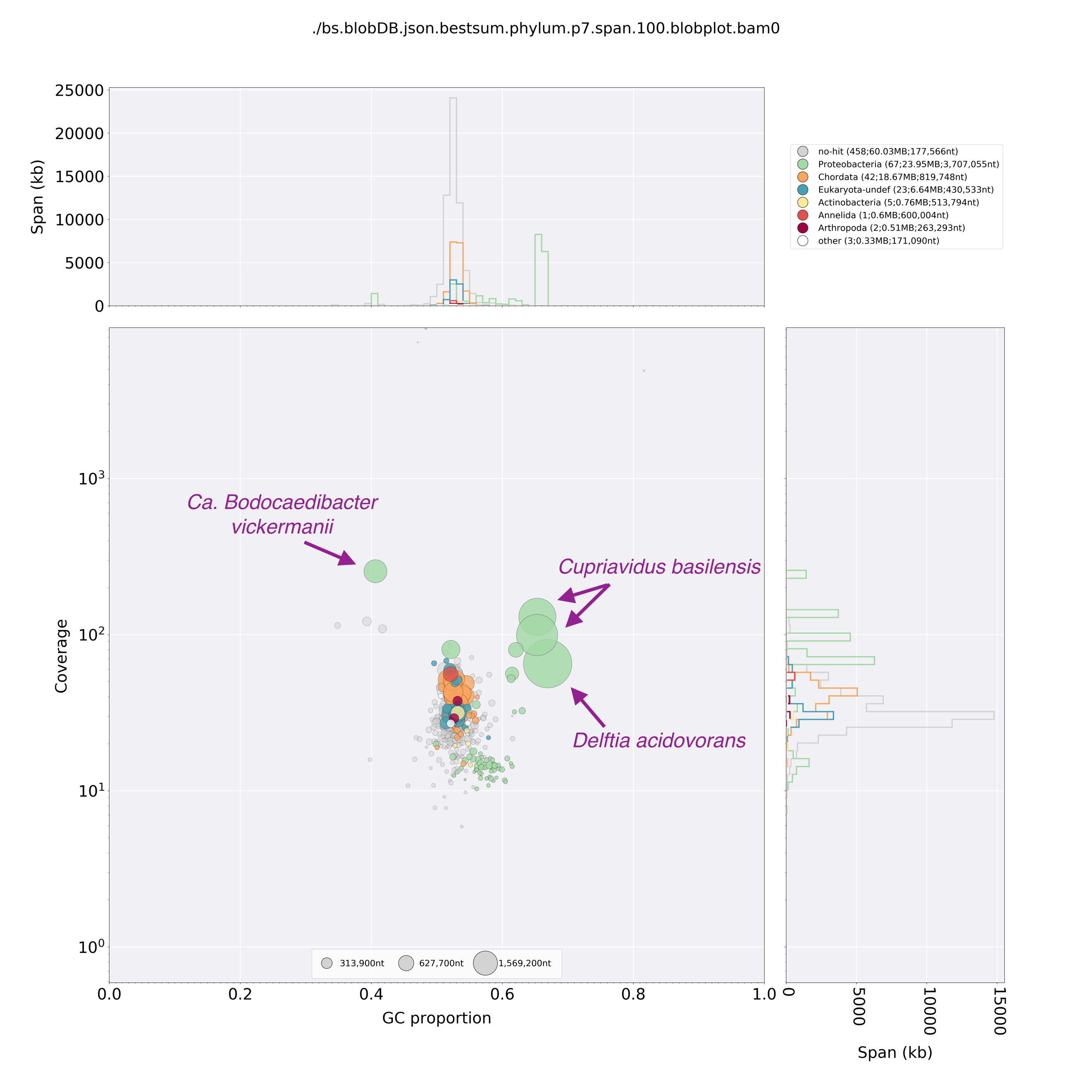


**Supplementary Figure 1: Blobplot analysis of genome assembly**

Analysis of genome assembly by mapping sequencing reads back to assembly and blast analysis against nt database of NCBI. Various contigs are plotted in the graph, with GC content on X-axis and coverage on Y-axis. Size of the circle is proportional to size of the contig. Different biological lineages are represented in different colours.


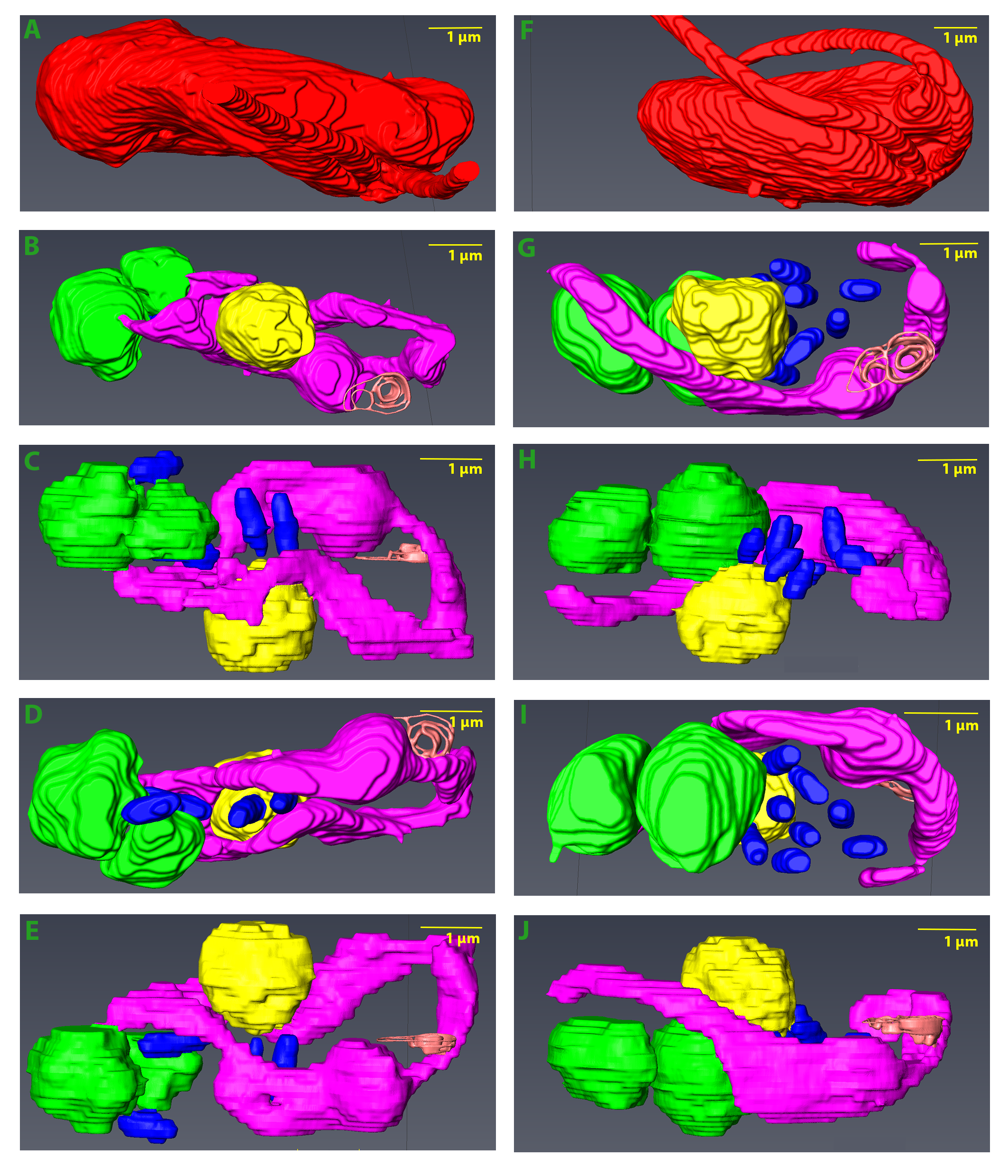


**Supplementary Figure 2: 3D model of *Bodo saltans* cell generated using Serial Block Face-Scanning Electron Microscopy.**

Animations of *B. saltans* cellular ultrastructure in longitudinal section. Two cells are shown with differences in endosymbiont number and distribution: cell 1 (A-E) and cell 2 (F-J). For each cell, one image with cell envelope (A, F) and four longitudinal sections with consecutive 90-degree rotation around radial axis are shown (B-E and G-J). Cell envelope (in red) shows the bean shaped structure of *B. saltans* and two flagella emerging from flagellar pocket (in orange). Nucleus (in yellow) is situated in the centre of the cell and cytobionts (in blue) are distributed in close vicinity to the nucleus. A large, swirled mitochondrion (in magenta) is placed around the periphery of the cell, with a kinetoplast capsule just under the flagellar pocket. Multiple food vacuoles (in green) are present at the posterior end of the cell.


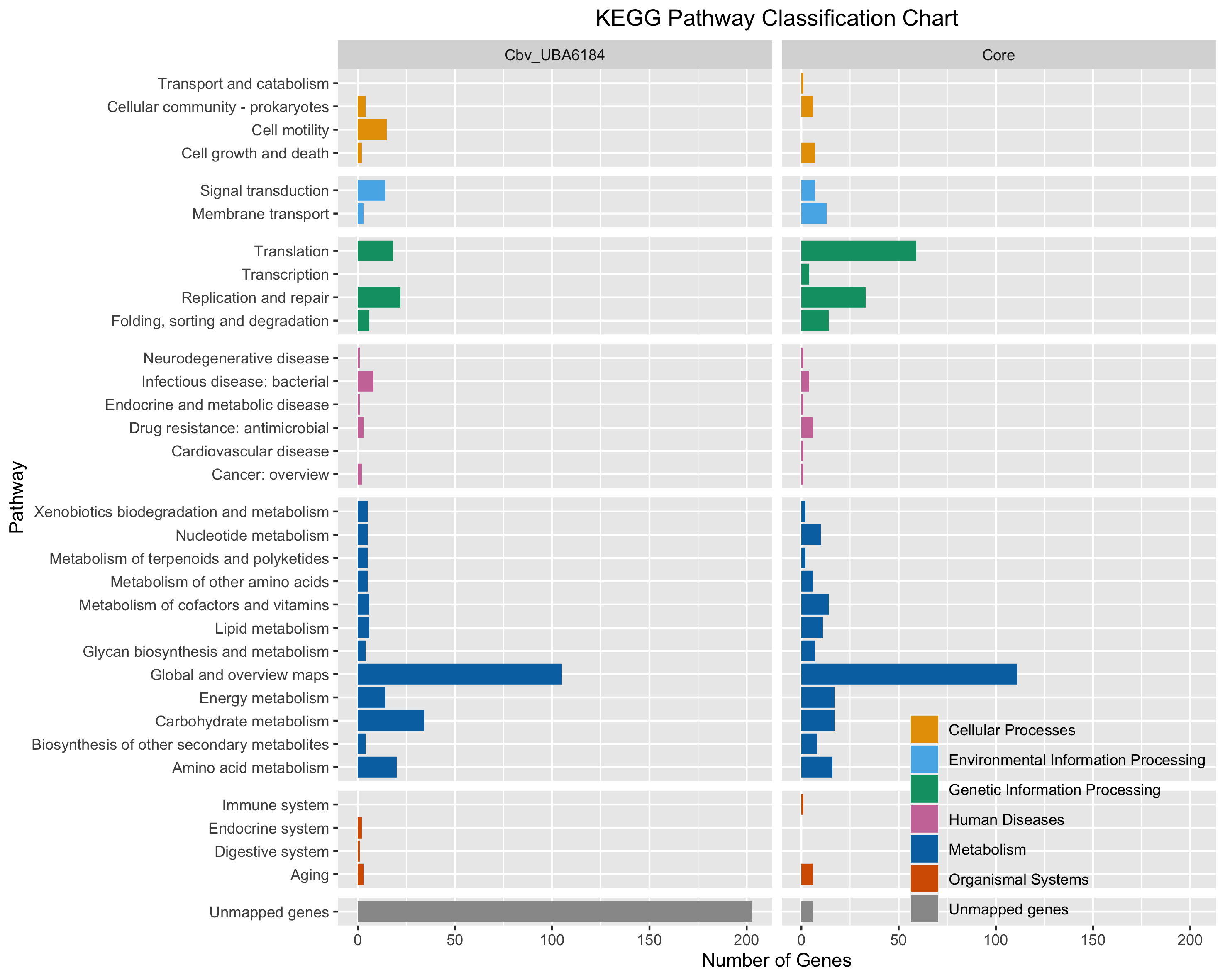


**Supplementary Figure 3: KEGG Pathway Classification Chart**

Based on the OrthoMCL analysis, core orthologues (shared by 13 protistan endosymbionts) and orthologues limited to *Candidatu*s Bodocaedibacter vickermanii (Cbv) and *Candidatus* Paracaedibacteraceae bacterium UBA6184 were identified. The bar graph shows the number of genes mapped on each pathway type on KEGG database. The colour of bars represents the six major pathway categories. Genes that did not map to any of the pathways are labelled as unmapped genes.


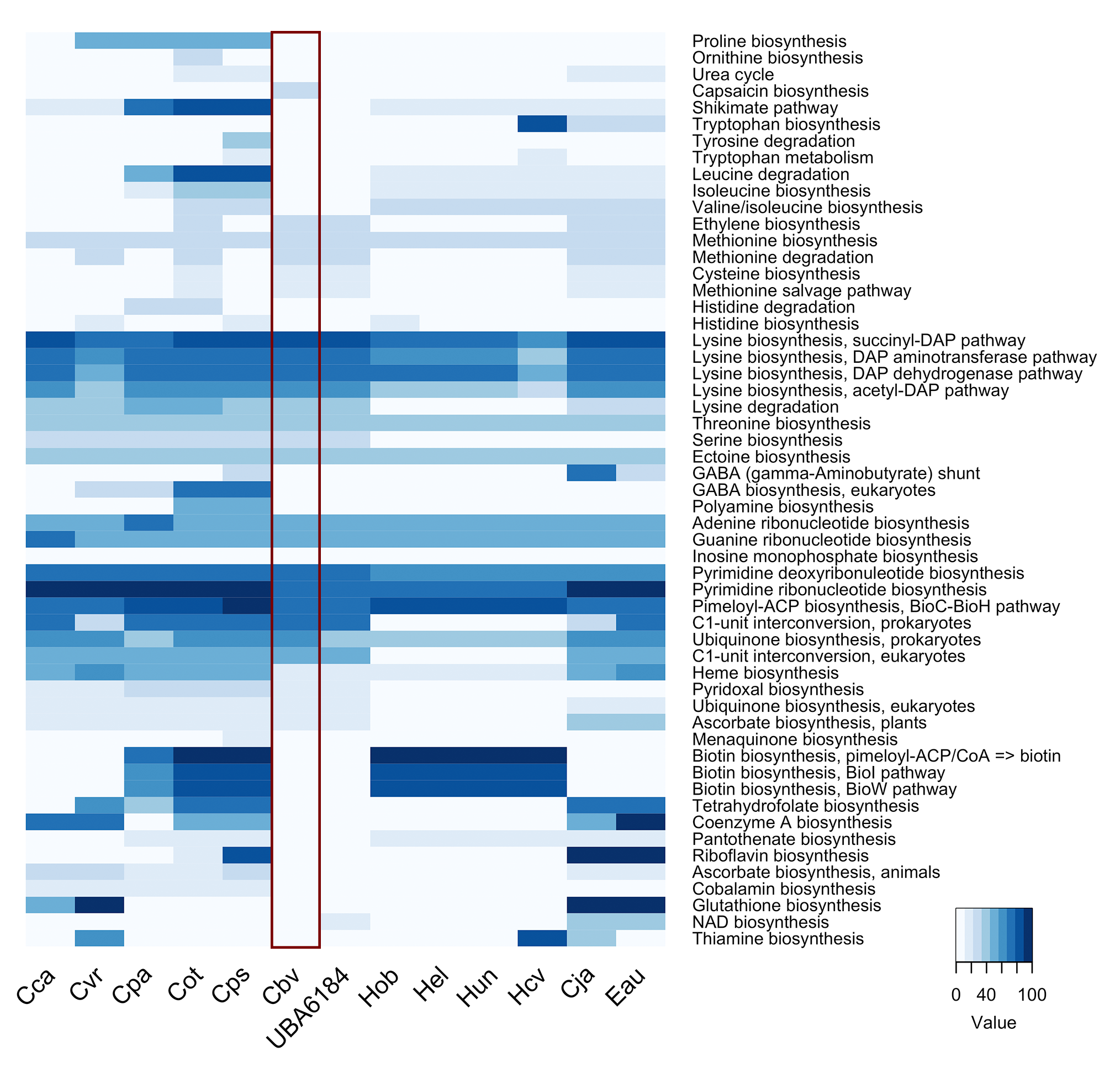


**Supplementary Figure 4: Comparison of metabolic pathways based on genome annotations for *Candidatus* Bodocaedibacter vickermanii and other alpha-proteobacterial endosymbionts of protists.**

Pathways involved in purine metabolism, pyrimidine metabolism, amino acid metabolism, polyamine biosynthesis, cofactor and vitamin biosynthesis are compared. The heatmap is based on MCR (Module completion ratio) values calculated using MAPLE v2.3.0. MCR is an indicator of completeness of a pathway in an organism. Based on the number of components of a pathway encoded by a genome, score varies from 0-100. MCR score of 100 means a complete metabolic pathway is encoded by the genome.


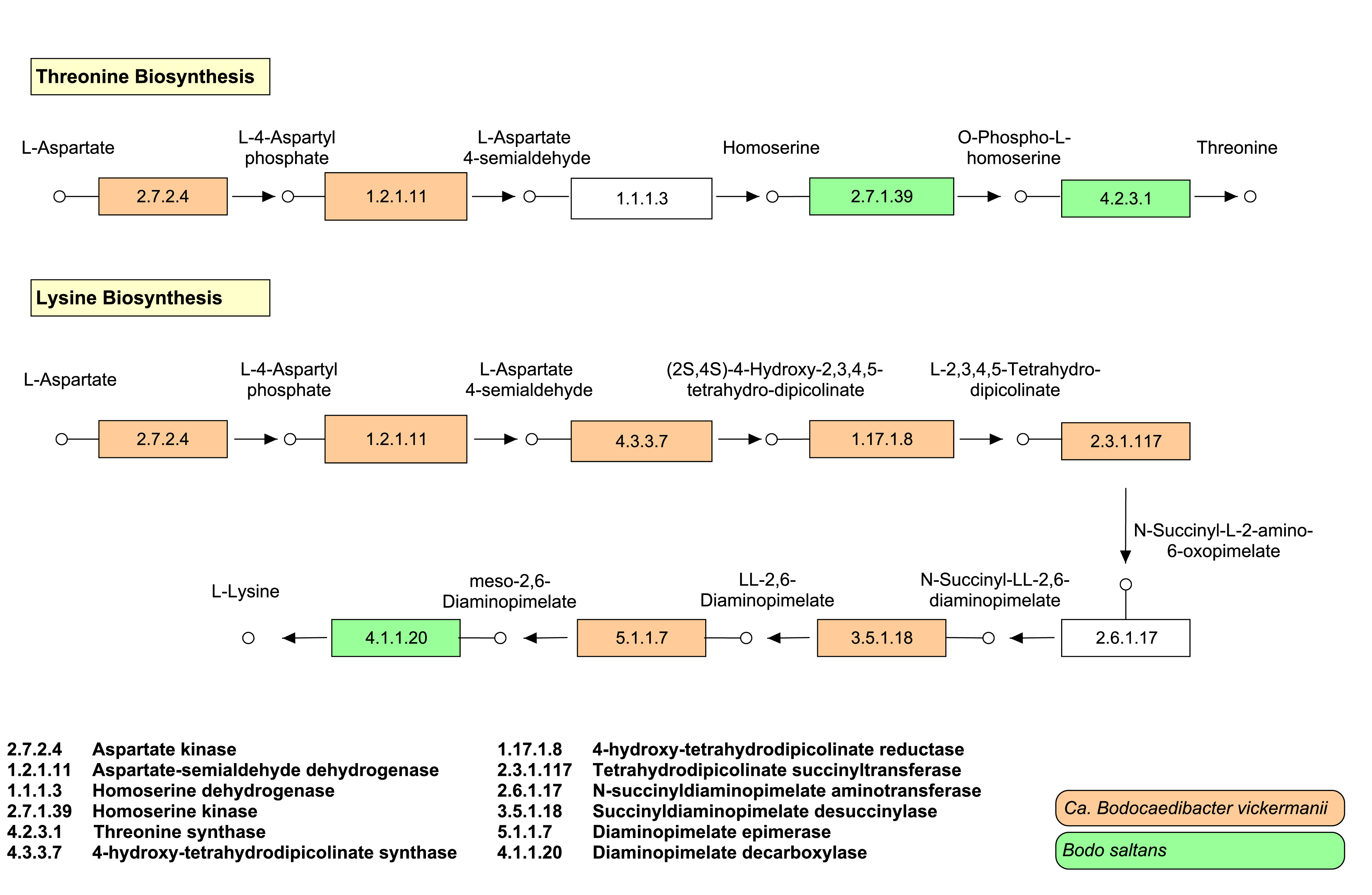


**Supplementary Figure 5: Amino acids biosynthesis pathways in *Candidatus* Bodocaedibacter vickermanii and *Bodo saltans***

Biosynthesis pathways for synthesis of L-lysine and threonine from L-aspartate are shown. Coloured boxes represent the presence of genes encoding for specific enzymes in *Ca.* B. vickermanii (in green) and *B. saltans* (in orange). White boxes represent the enzymes for which encoding genes could not be found in both organisms.


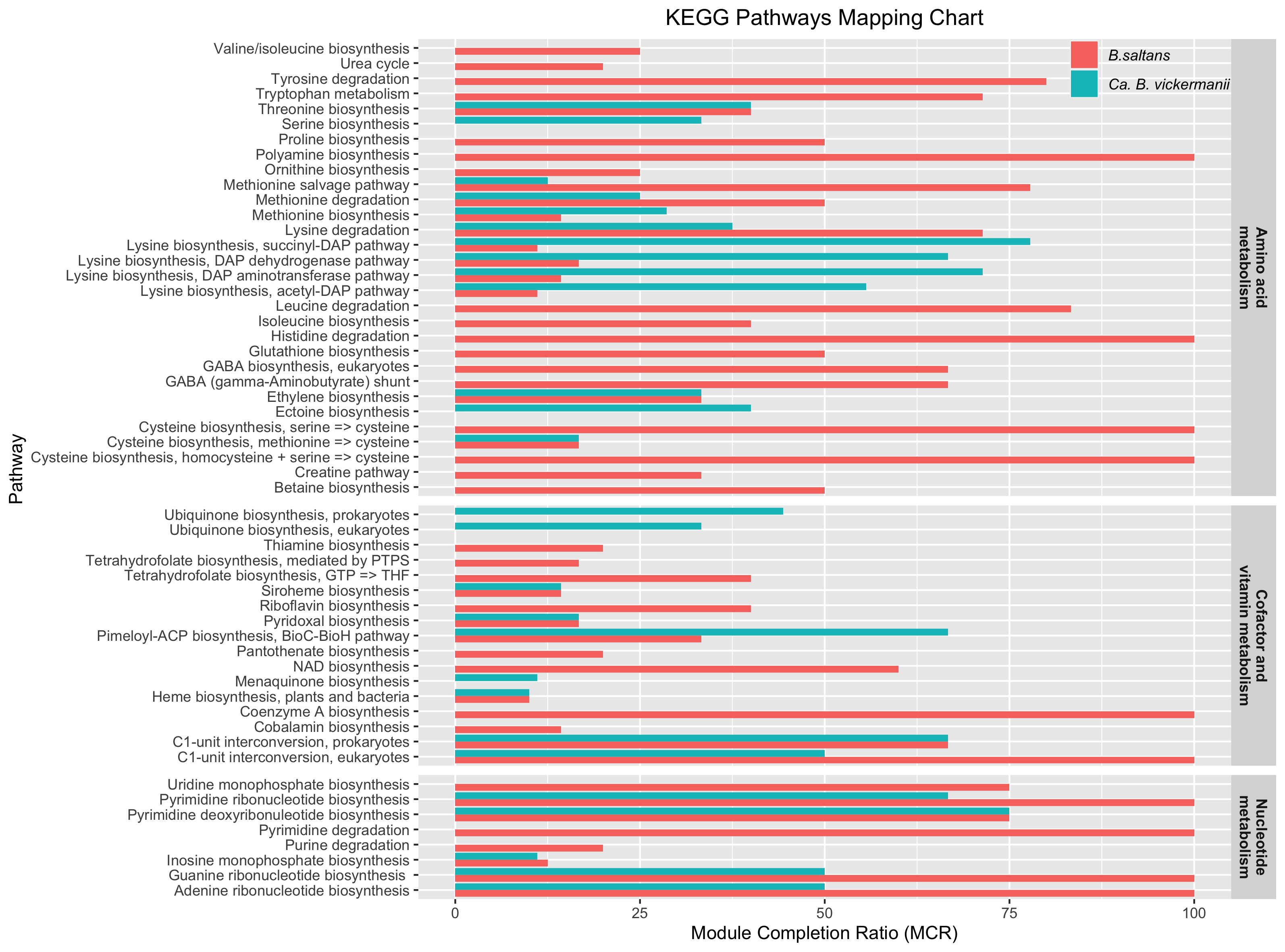


**Supplementary Figure 6: KEGG Pathway Classification Chart**

Pathways belonging to nucleotide metabolism, amino acid metabolism and cofactor and vitamins metabolism are compared between *Bodo saltans* and its endosymbiont, *Candidatu*s Bodocaedibacter vickermanii. The bar graph is based on MCR (Module completion ratio) values calculated using Genomaple v2.3.2. MCR is an indicator of completeness of a pathway in an organism. Based on the number of components of a pathway encoded by a genome, score varies from 0-100. MCR score of 100 means a complete metabolic pathway is encoded by the genome.


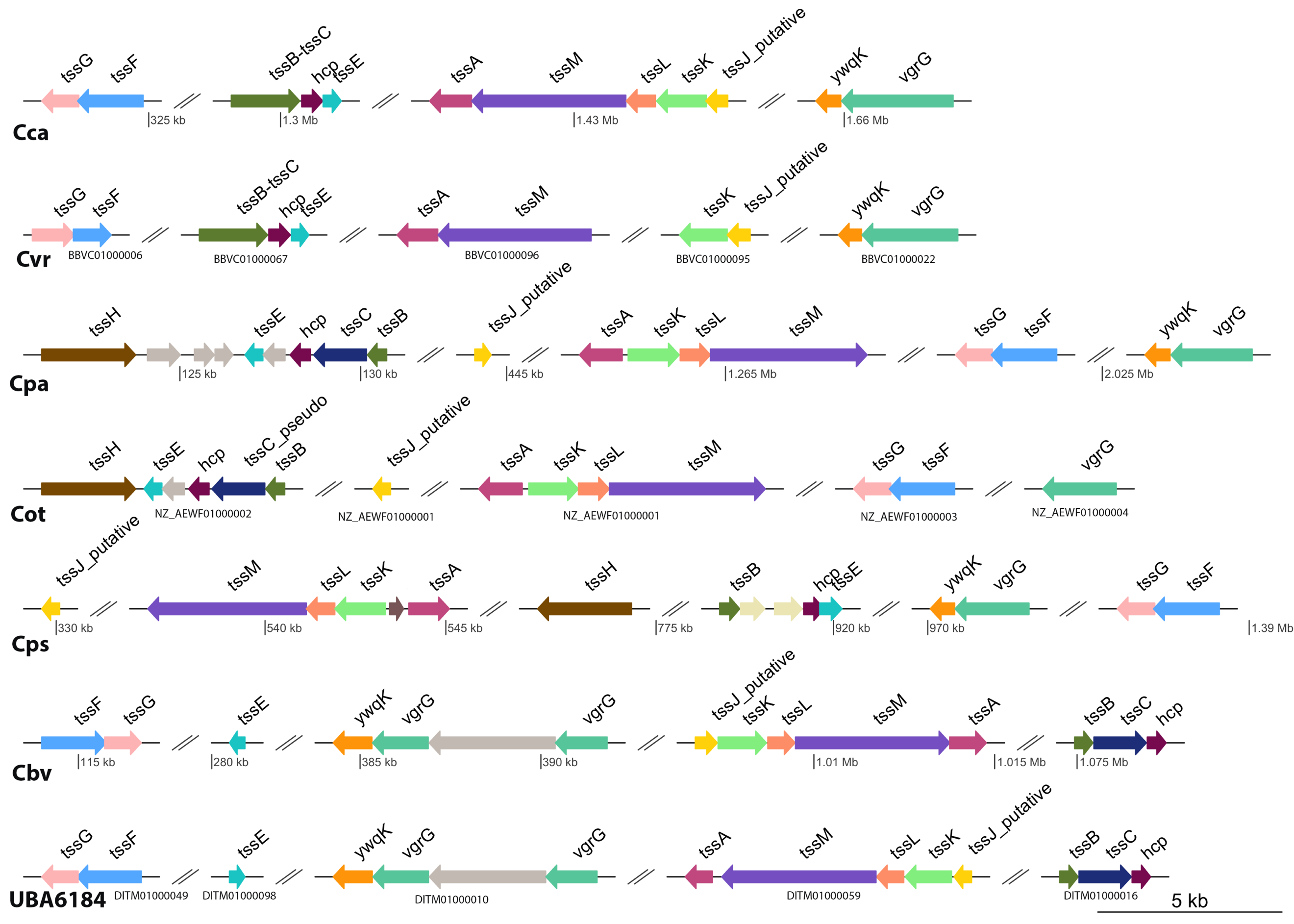


**Supplementary Figure 7: Schematic structure of Type VI Secretion System cluster in *Candidatus* Bodocaedibacter vickermanii and other alpha-proteobacterial endosymbionts of Paracedibacteraceae and Caedimonadaceae.**

T6SS core genes are dispersed at four or more locations in the genome. All maps are on the scale. Legends in grey show the location in chromosome (complete genomes) or the contig accession number (draft genomes).


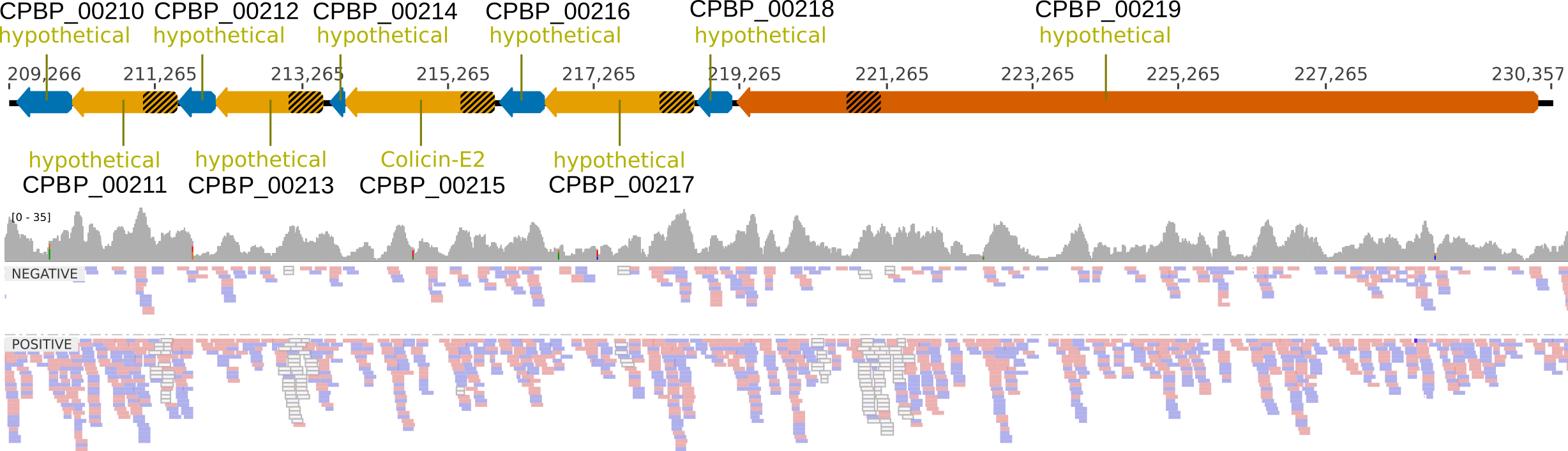
**A) Polymorphic Toxin System I**

**B) Polymorphic Toxin System II**


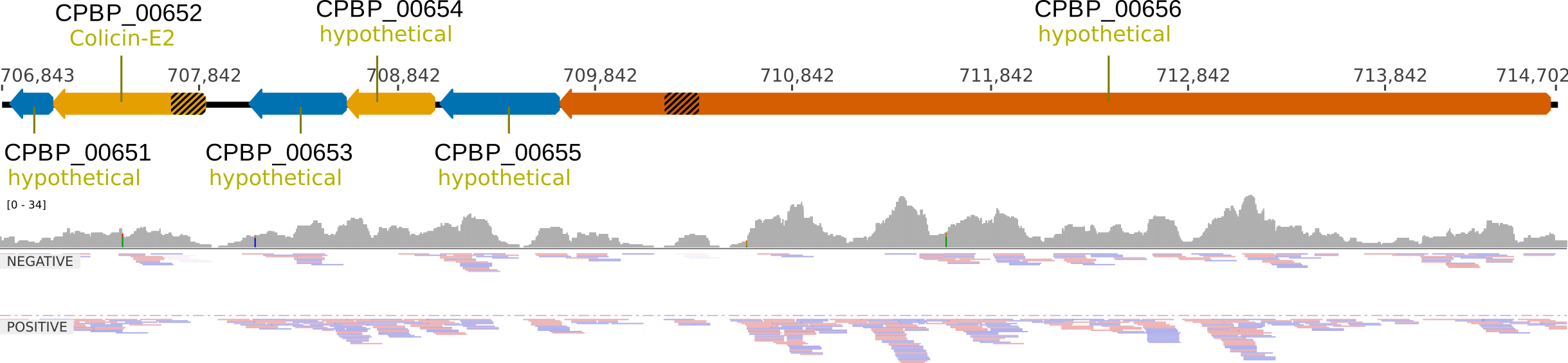


**C) Polymorphic Toxin System III**


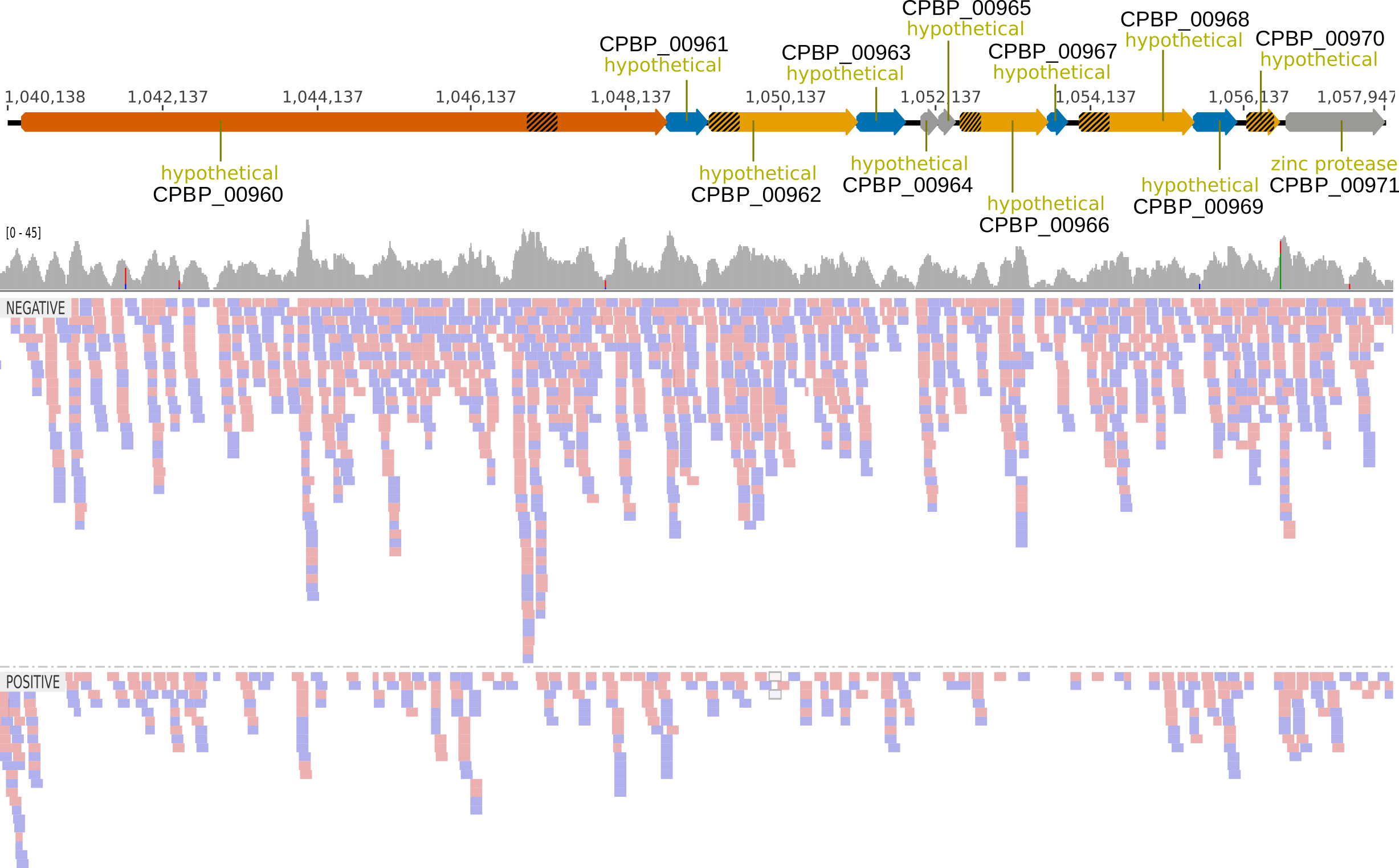


**Supplementary Figure 8: Polymorphic toxin systems in *Ca.* B. vickermanii and gene expression**

Schematic diagrams of the organization of the three predicted polymorphic toxin systems in *Ca*. B. vickermanii. RNA-seq reads are mapped on various genes and strand-specific reads quantified by SeqMonk program are shown separately.
