# Supplementary material for: Bodo saltans (Kinetoplastida) is dependent on a novel Paracaedibacter-like endosymbiont that possesses multiple putative toxin-antitoxin systems
Source: ISME J. 2021 Jan 15;15(6):1680–94. doi: 10.1038/s41396-020-00879-6 (PMC8163844; doi:10.1038/s41396-020-00879-6)
Supplement: Supplementary file 2 — Supplementary Table 1 [file 41396_2020_879_MOESM2_ESM.pdf]

Supplementary Table 1: Prevalence of *Candidatus* Bodocaedibacter vickermanii in diverse environments based on 16S rRNA gene at different similarity thresholds (99, 97, 95 and 90%)

**Origin**	**Sample-Number**	**Prevalence (numbers)**				**Prevalence (percentage)**			
		**99**	**97**	**95**	**90**	**99**	**97**	**95**	**90**
freshwater metagenome	13532	619	1893	2671	3235	4.57	13.99	19.74	23.91
lake water metagenome	376	17	72	128	136	4.52	19.15	34.04	36.17
riverine metagenome	277	15	31	33	37	5.42	11.19	11.91	13.36
wastewater metagenome	2451	72	589	681	741	2.94	24.03	27.78	30.23
activated sludge metagenome	1370	74	226	270	300	5.40	16.50	19.71	21.90
aquatic metagenome	10177	308	844	1286	1629	3.03	8.29	12.64	16.01
marine metagenome	33683	107	396	729	1951	0.32	1.18	2.16	5.79
freshwater sediment metagenome	1337	3	18	26	34	0.22	1.35	1.94	2.54
marine sediment metagenome	2396	0	3	11	35	0.00	0.13	0.46	1.46
soil crust metagenome	165	2	3	3	8	1.21	1.82	1.82	4.85
soil metagenome	57972	610	1929	2518	3200	1.05	3.33	4.34	5.52
Human gut metagenome	36953	10	17	19	20	0.03	0.05	0.05	0.05
plant metagenome	10679	111	663	904	1005	1.04	6.21	8.47	9.41
rhizosphere metagenome	13304	686	2033	2224	2443	5.16	15.28	16.72	18.36
