# Supplementary material for: Bodo saltans (Kinetoplastida) is dependent on a novel Paracaedibacter-like endosymbiont that possesses multiple putative toxin-antitoxin systems
Source: ISME J. 2021 Jan 15;15(6):1680–94. doi: 10.1038/s41396-020-00879-6 (PMC8163844; doi:10.1038/s41396-020-00879-6)
Supplement: Supplementary file 3 — Supplementary Table 2 [file 41396_2020_879_MOESM3_ESM.pdf]

Supplementary Table 2: List of genes in *Candidatus* Bodocaedibacter vickermanii encoding for transporters, BLAST hits and TCDB classification

Gene_ID	Length (amino acids)	Best Blast Hit (genBank Accession Number)	E-value	Transport Classification Database (TCDB) Family		Number of transmenbrane α-helices
				Family ID	Family name	
CPBP_00565	516	ATP/ADP translocase (Fragment) Lawsonia intracellularis (B0RZB7)	1.0E-172	2.A.12.	The ATP:ADP Antiporter (AAA) Family	10
CPBP_00255	389	Proton/Sodium-glutamate symport protein Bacillus stearothermophilus (P24943)	1.3E-11	2.A.23.	The Dicarboxylate/Amino Acid:Cation (Na+ or H+) Symporter (DAACS) Family	9
CPBP_00720	400	Excitatory amino acid transporter 1 (Sodium dependent glutamate/aspartate transporter 1) Rattus norvegicus (P24942)	7.4E-12	2.A.23.	The Dicarboxylate/Amino Acid:Cation (Na+ or H+) Symporter (DAACS) Family	10
CPBP_00377	381	Branched-chain amino acid transport system carrier protein brnQ Chlamydia trachomatis (O84558)	1.5E-62	2.A.26.	The Branched Chain Amino Acid:Cation Symporter (LIVCS) Family	11
CPBP_00197	434	Arginine/agmatine antiporter Escherichia coli (P60061)	3.8E-74	2.A.3.	The Amino Acid-Polyamine-Organocation (APC) Family	11
CPBP_00879	402	Tyrosine-specific transport protein Escherichia coli (P0AAD4)	1.7E-101	2.A.42.	The Hydroxy/Aromatic Amino Acid Permease (HAAAP) Family	11
CPBP_00324	300	Uncharacterized transporter HP_1234 Helicobacter pylori (O25832)	5.2E-25	2.A.7	The Drug/Metabolite Transporter (DMT) Superfamily	10
CPBP_01233	271	Hypothetical protein RP076 Rickettsia prowazekii (Q9ZE70)	1.4E-17	2.A.7.	The Drug/Metabolite Transporter (DMT) Superfamily	9

CPBP_00271	456	Sodium/Pantothenate symporter (Pantothenate permease) Escherichia coli (P16256)	4.8E-23	2.A.21.	The Solute:Sodium Symporter (SSS) Family	13
CPBP_01198	501	Phenylacetic acid permease Pseudomonas putida (O50471)	3.6E-163	2.A.21.	The Solute:Sodium Symporter (SSS) Family	13
CPBP_00044	227	Major facilitator family transporter Legionella pneumophila (Q5ZUB4)	1.1E-38	2.A.1.	The Major Facilitator Superfamily (MFS)	6
CPBP_00045	189	Major facilitator family transporter Legionella pneumophila (Q5ZUB4)	4.1E-37	2.A.1.	The Major Facilitator Superfamily (MFS)	5
CPBP_00198	404	Bicyclomycin resistance protein (Sulfonamide resistance protein) Escherichia coli (P28246)	1.4E-39	2.A.1.	The Major Facilitator Superfamily (MFS)	12
CPBP_00206	444	Putative haloacid permease Burkholderia cepacia (Q7X4L6)	4.8E-30	2.A.1.	The Major Facilitator Superfamily (MFS)	10
CPBP_00232	432	Major facilitator superfamily MFS_1 Nostoc punctiforme (B2JBG5)	2.6E-64	2.A.1.	The Major Facilitator Superfamily (MFS)	10
CPBP_00250	400	Bicyclomycin resistance protein Escherichia coli (P28246)	7.4E-41	2.A.1.	The Major Facilitator Superfamily (MFS)	12
CPBP_00330	419	Proline/betaine transporter Escherichia coli (P0C0L7)	5.3E-78	2.A.1.	The Major Facilitator Superfamily (MFS)	12
CPBP_00368	1024	2-acylglycerophosphoethanolamine acyltransferase Bradyrhizobium japonicum (Q89SS6)	1.4E-57	2.A.1.	The Major Facilitator Superfamily (MFS)	13
CPBP_00437	404	Bicyclomycin resistance protein Escherichia coli (P28246)	2.1E-31	2.A.1.	The Major Facilitator Superfamily (MFS)	12
CPBP_00467	436	Putative Major facilitator family transporter Legionella pneumophila (I7I571)	4.9E-70	2.A.1.	The Major Facilitator Superfamily (MFS)	12
CPBP_00469	399	Protein ampG Escherichia coli (P0AE16)	1.6E-45	2.A.1.	The Major Facilitator Superfamily (MFS)	12

CPBP_00470	414	Putative signal transducer protein Neisseria gonorrhoeae (Q5F6G0)	6.6E-52	2.A.1.	The Major Facilitator Superfamily (MFS)	10
CPBP_00549	417	Putative Major facilitator family transporter Legionella pneumophila (I7I0I4)	9.4E-52	2.A.1.	The Major Facilitator Superfamily (MFS)	12
CPBP_00557	264	Putative to nasA protein Legionella pneumophila (I7I1R6)	1.4E-55	2.A.1.	The Major Facilitator Superfamily (MFS)	6
CPBP_00558	163	Putative to nasA protein Legionella pneumophila (I7I1R6)	6.9E-16	2.A.1.	The Major Facilitator Superfamily (MFS)	4
CPBP_00559	397	Bicyclomycin resistance protein Escherichia coli (P28246)	3.2E-36	2.A.1.	The Major Facilitator Superfamily (MFS)	12
CPBP_00586	422	Major facilitator superfamily MFS_1 Nostoc punctiforme (B2JBG5)	2.6E-71	2.A.1.	The Major Facilitator Superfamily (MFS)	10
CPBP_00795	464	Glycerol-3-phosphate transpoter (GlpT) Rickettsia prowazekii (Q9ZE92)	9.9E-97	2.A.1.	The Major Facilitator Superfamily (MFS)	12
CPBP_00947	413	Glycerol-3-phosphate transpoter (GlpT) Rickettsia prowazekii (Q9ZE92)	1.1E-106	2.A.1.	The Major Facilitator Superfamily (MFS)	10
CPBP_01035	416	Putative Major facilitator family transporter Legionella pneumophila (I7HQW2)	8.9E-60	2.A.1.	The Major Facilitator Superfamily (MFS)	12
CPBP_01108	458	Proline/betaine transporter Escherichia coli (P0C0L7)	6.5E-30	2.A.1.	The Major Facilitator Superfamily (MFS)	10
CPBP_01110	421	Inner membrane metabolite transport protein yhjE Escherichia coli (P37643)	2.1E-63	2.A.1.	The Major Facilitator Superfamily (MFS)	12
CPBP_00267	276	Regulator of acetyl-CoA synthetase activity Saccharomyces cerevisiae (P33303)	2.0E-13	2.A.29.	The Mitochondrial Carrier (MC) Family	2
CPBP_00034	284	ABC transporter permease protein Streptococcus pyogenes (Q99ZY4)	1.4E-38	3.A.1.	The ATP-binding Cassette (ABC) Superfamily	7
CPBP_00035	249	Putative ABC transporter (ATP-binding protein) Streptococcus pyogenes (Q99ZY3)	1.0E-51	3.A.1.	The ATP-binding Cassette (ABC) Superfamily	0

CPBP_00036	317	BC transporter substrate-binding protein Streptococcus pyogenes (Q99ZY6)	5.5E-23	3.A.1.	The ATP-binding Cassette (ABC) Superfamily	0
CPBP_00040	945	ABC transporter related Chloroflexus aurantiacus (A9WBR9)	4.4E-11	3.A.1.	The ATP-binding Cassette (ABC) Superfamily	0
CPBP_00058	251	Zinc import ATP-binding protein znuC Escherichia coli (P0A9X1)	3.1E-58	3.A.1.	The ATP-binding Cassette (ABC) Superfamily	0
CPBP_00059	264	High-affinity zinc uptake system membrane protein znuB Escherichia coli (P39832)	2.1E-26	3.A.1.	The ATP-binding Cassette (ABC) Superfamily	7
CPBP_00106	594	ABC transporter-related protein Ralstonia metallidurans (Q1LRE9)	0.0E+00	3.A.1.	The ATP-binding Cassette (ABC) Superfamily	6
CPBP_00140	525	OptrA Enterococcus faecalis (ANC59923)	1.5E-99	3.A.1.	The ATP-binding Cassette (ABC) Superfamily	0
CPBP_00180	235	Lipoprotein releasing system ATP-binding protein lolD Escherichia coli (P75957)	2.8E-59	3.A.1.	The ATP-binding Cassette (ABC) Superfamily	0
CPBP_00181	416	Lipoprotein releasing system transmembrane protein lolE Escherichia coli (P75958)	3.7E-50	3.A.1.	The ATP-binding Cassette (ABC) Superfamily	4
CPBP_00283	614	ABC transporter Acetobacter aceti (Q2PGB8)	6.8E-134	3.A.1.	The ATP-binding Cassette (ABC) Superfamily	0
CPBP_00298	338	ABC transporter substrate-binding protein Streptococcus pyogenes (Q99ZY6)	8.7E-40	3.A.1.	The ATP-binding Cassette (ABC) Superfamily	1
CPBP_00307	560	Energy-dependent translational throttle protein EttA Mycobacterium tuberculosis (P9WQK3)	0.0E+00	3.A.1.	The ATP-binding Cassette (ABC) Superfamily	0
CPBP_00477	260	Predicted ATPase involved in cell division Caldanaerobacter subterraneus (Q8R8L8)	2.1E-47	3.A.1.	The ATP-binding Cassette (ABC) Superfamily	0
CPBP_00583	150	Probable phospholipid ABC transporter-binding protein mlaD Escherichia coli (P64604)	3.3E-15	3.A.1.	The ATP-binding Cassette (ABC) Superfamily	1

CPBP_00784	371	Putrescine-binding periplasmic protein precursor<br>Escherichia coli (P31133)	1.4E-49	3.A.1.	The ATP-binding Cassette (ABC) Superfamily	1
CPBP_00785	375	Putrescine transport ATP-binding protein potG<br>Escherichia coli (P31134)	3.0E-125	3.A.1.	The ATP-binding Cassette (ABC) Superfamily	0
CPBP_00786	311	Putrescine transport system permease protein potH<br>Escherichia coli (P31135)	2.0E-103	3.A.1.	The ATP-binding Cassette (ABC) Superfamily	6
CPBP_00787	269	Putrescine transport system permease protein potI<br>Escherichia coli (P0AFL1)	5.5E-82	3.A.1.	The ATP-binding Cassette (ABC) Superfamily	6
CPBP_00805	240	Probable amino-acid ABC transporter ATP-binding protein yqiZ<br>Bacillus subtilis (P54537)	5.0E-97	3.A.1.	The ATP-binding Cassette (ABC) Superfamily	0
CPBP_00806	218	Probable amino-acid ABC transporter permease protein yqiY<br>Bacillus subtilis (P54536)	8.2E-65	3.A.1.	The ATP-binding Cassette (ABC) Superfamily	3
CPBP_00807	256	Probable amino-acid ABC transporter extracellular-binding protein yqiX<br>Bacillus subtilis (P54535)	2.6E-36	3.A.1.	The ATP-binding Cassette (ABC) Superfamily	0
CPBP_00918	253	Hypothetical protein At1g19800 (Permease-like protein)<br>Arabidopsis thaliana (Q8L4R0)	2.3E-46	3.A.1.	The ATP-binding Cassette (ABC) Superfamily	5
CPBP_00919	252	Putative ATPase component of ABC transporter system<br>Sphingobium japonicum (A4PCH8)	2.1E-53	3.A.1.	The ATP-binding Cassette (ABC) Superfamily	0
CPBP_01091	269	High-affinity zinc uptake system protein znuA precursor<br>Escherichia coli (P39172)	5.6E-21	3.A.1.	The ATP-binding Cassette (ABC) Superfamily	0
CPBP_01190	596	Mitochondrial ATP-binding cassette 2<br>Homo sapiens (Q9NRK6)	1.8E-108	3.A.1.	The ATP-binding Cassette (ABC) Superfamily	5
CPBP_01236	519	Mitochondrial transpoter ATM1<br>Rickettsia prowazekii (Q9ZDW0)	3.2E-30	3.A.1.	The ATP-binding Cassette (ABC) Superfamily	5
CPBP_00691	315	Putative uncharacterized protein<br>Hoeflea phototrophica (A9DHE6)	2.4E-52	2.A.83.	The Na+-dependent Bicarbonate Transporter (SBT) Family	8

CPBP_00050	178	BioY family protein<br>Rickettsia typhi (Q68X47)	7.7E-20	2.A.88.	The Vitamin Uptake Transporter (VUT or ECF) Family	5
CPBP_00587	1030	Probable Resistance-Nodulation- Cell Division (RND) efflux transporter<br>Pseudomonas aeruginosa (Q9HW27)	0.0E+00	2.A.6.	The Resistance-Nodulation-Cell Division (RND) Superfamily	12
CPBP_00588	382	Probable RND efflux membrane- fusion protein<br>Burkholderia glumae (Q4VSJ3)	2.9E-41	2.A.6.	The Resistance-Nodulation-Cell Division (RND) Superfamily	1
CPBP_00823	534	Protein-export membrane protein secD<br>Escherichia coli (P0AG90)	6.1E-86	2.A.6.	The Resistance-Nodulation-Cell Division (RND) Superfamily	5
CPBP_00824	310	Protein translocase subunit SecF<br>Escherichia coli (P0AG93)	5.6E-55	2.A.6.	The Resistance-Nodulation-Cell Division (RND) Superfamily	6
CPBP_00861	367	Multidrug efflux transporter VexE<br>Vibrio cholerae (A6P7H2)	6.5E-47	2.A.6.	The Resistance-Nodulation-Cell Division (RND) Superfamily	1
CPBP_00863	1055	Multidrug efflux transporter VexF<br>Vibrio cholerae (A6P7H3)	0.0E+00	2.A.6.	The Resistance-Nodulation-Cell Division (RND) Superfamily	12
CPBP_00312	514	Virulence factor mviN<br>Salmonella typhimurium (P37169)	1.2E-66	2.A.66.	The Multidrug/Oligosaccharidyl-lipid/Polysaccharide (MOP) Flippase Superfamily	13
CPBP_00313	524	Virulence factor mviN<br>Salmonella typhimurium (P37169)	3.6E-73	2.A.66.	The Multidrug/Oligosaccharidyl-lipid/Polysaccharide (MOP) Flippase Superfamily	12
CPBP_00898	455	Probable multidrug resistance protein NorM<br>Thermotoga maritima (Q9WZS2)	2.3E-14	2.A.66.	The Multidrug/Oligosaccharidyl-lipid/Polysaccharide (MOP) Flippase Superfamily	12
CPBP_00899	452	Probable multidrug resistance protein NorM<br>Thermotoga maritima (Q9WZS2)	2.4E-19	2.A.66.	The Multidrug/Oligosaccharidyl-lipid/Polysaccharide (MOP) Flippase Superfamily	12
CPBP_00207	315	Na+/H+ antiporter<br>Vibrio parahaemolyticus (Q56725)	1.6E-67	2.A.33.	The NhaA Na+:H+ Antiporter (NhaA) Family	9
CPBP_00571	441	Na+/H+ antiporter family protein<br>Shewanella oneidensis (Q8EHX2)	1.7E-127	2.A.35.	The NhaC Na+:H+ Antiporter (NhaC) Family	9

CPBP_00707	402	K(+) efflux antiporter 2, chloroplastic Arabidopsis thaliana (O65272)	1.1E-91	2.A.37.	The Monovalent Cation:Proton Antiporter-2 (CPA2) Family	11
CPBP_00342	458	Trk system potassium uptake protein trkA Escherichia coli (P0AGI8)	2.4E-69	2.A.38	The K+ Transporter (Trk) Family	0
CPBP_00990	483	Trk system potassium uptake protein TrkI Halomonas elongata (Q6T3V6)	9.3E-112	2.A.38.	The K+ Transporter (Trk) Family	12
CPBP_00433	694	H+ translocating pyrophosphate synthase Rhodospirillum rubrum (O68460)	0.0E+00	3.A.10.	The H+-translocating Pyrophosphatase (H+-PPase) Family	13
CPBP_00608	492	NADH dehydrogenase 1, chain 14 Paracoccus denitrificans (P29926)	6.5E-74	3.D.1.	The Proton-translocating NADH Dehydrogenase (NDH) Family	12
CPBP_00609	490	NADH dehydrogenase 1, chain 13 Paracoccus denitrificans (P29925)	7.2E-135	3.D.1.	The Proton-translocating NADH Dehydrogenase (NDH) Family	14
CPBP_00610	641	NADH dehydrogenase 1, chain 12 Paracoccus denitrificans (P29924)	9.4E-155	3.D.1.	The Proton-translocating NADH Dehydrogenase (NDH) Family	16
CPBP_00614	335	NADH dehydrogenase 1, chain 8 Paracoccus denitrificans (P29920)	3.2E-105	3.D.1.	The Proton-translocating NADH Dehydrogenase (NDH) Family	8
CPBP_00134	264	Ion transport protein Arcobacter butzleri (A8EVM5)	8.2E-33	1.A.1.	The Voltage-gated Ion Channel (VIC) Superfamily	6
CPBP_00358	426	Mg2+ and Co2+ transporter CorB, contains DUF21, CBS pair, and CorC-HlyC domains Pseudomonas bauzanensis (SES26846)	1.1E-67	1.A.112	The Cyclin M Mg2+ Exporter (CNNM) Family	3
CPBP_00231	458	Magnesium transporter MgtE Prochlorococcus marinus (A2C579)	7.0E-50	1.A.26	The Mg2+ Transporter-E (MgtE) Family	5
CPBP_01170	142	Biopolymer transport protein exbD Escherichia coli (P0ABV2)	1.0E-30	1.A.30	The H+- or Na+-translocating Bacterial Flagellar Motor/ExbBD Outer Membrane Transport Energizer (Mot-Exb) Superfamily	1
CPBP_01171	223	Protein tolQ Escherichia coli (P0ABU9)	1.8E-52	1.A.30	The H+- or Na+-translocating Bacterial Flagellar Motor/ExbBD Outer Membrane Transport Energizer (Mot-Exb) Superfamily	3

CPBP_00693	547	Membrane protein, UPF0126<br>Gramella forsetii (A0M015)	3.9E-11	1.A.62.	The Homotrimeric Cation Channel (TRIC) Family	7
CPBP_00694	539	Uncharacterized protein<br>Yersinia pestis (I7MT28)	1.9E-12	1.A.62.	The Homotrimeric Cation Channel (TRIC) Family	7
CPBP_01086	482	Putative outer membrane secretion protein<br>Rhizobium meliloti (Q92Q38)	2.4E-26	1.B.17.	The Outer Membrane Factor (OMF) Family	0
CPBP_01082	276	Hemolysin C<br>Brachyspira hyodysenteriae (Q54318)	2.9E-36	1.C.126.	The HlyC (HlyC) Family of Haemolysins	0
CPBP_01232	295	Ferrous-iron efflux pump fieF<br>Escherichia coli (P69380)	6.1E-62	2.A.4.	The Cation Diffusion Facilitator (CDF) Family	5
CPBP_00925	369	Permease<br>Brucella abortus (C9VRY8)	1.6E-67	2.A.86.	The Autoinducer-2 Exporter (AI-2E) Family	8
CPBP_01020	561	Inner membrane protein YidC<br>Escherichia coli (P25714)	4.9E-60	2.A.9.	The Cytochrome Oxidase Biogenesis (Oxa1) Family	5
CPBP_00098	445	Preprotein translocase subunit secY<br>Escherichia coli (P0AGA2)	2.9E-140	3.A.5.	The General Secretory Pathway (Sec) Family	10
CPBP_00794	652	4-coumarate--CoA ligase 1<br>Arabidopsis thaliana (Q42524)	2.9E-06	4.C.1.	The Proposed Fatty Acid Transporter (FAT) Family	0
CPBP_00837	587	Ferrous iron transport protein B<br>Leptospira biflexa (Q5XPH7)	6.0E-87	9.A.8.	The Ferrous Iron Uptake (FeoB) Family	9
