# Supplementary material for: Bodo saltans (Kinetoplastida) is dependent on a novel Paracaedibacter-like endosymbiont that possesses multiple putative toxin-antitoxin systems
Source: ISME J. 2021 Jan 15;15(6):1680–94. doi: 10.1038/s41396-020-00879-6 (PMC8163844; doi:10.1038/s41396-020-00879-6)
Supplement: Supplementary file 4 — Supplementary Table 3 [file 41396_2020_879_MOESM4_ESM.pdf]

Supplementary Table 3: List of genes in *Candidatus* Bodocaedibacter vickermanii and their expression analysis

		gene length	Read count	RPK values
**Type VI Secretion System**				
**CPBP_00125**	Type VI secretion system baseplate subunit TssF	1785	21	11.8
**CPBP_00126**	Type VI secretion system baseplate subunit TssG	1017	36	35.4
**CPBP_00258**	type VI secretion system baseplate subunit TssE	432	12	27.8
**CPBP_00353**	type VI secretion system tip protein VgrG	1545	62	40.1
**CPBP_00355**	type VI secretion system tip protein VgrG	1434	43	30.0
**CPBP_00565**	ADP,ATP carrier protein 1	1551	214	138.0
**CPBP_00933**	type VI secretion system protein, membrane lipoprotein	615	18	29.3
**CPBP_00934**	type VI secretion system baseplate subunit TssK	1341	32	23.9
**CPBP_00935**	type VI secretion system protein TssL (DotU family)	759	18	23.7
**CPBP_00936**	type VI secretion system membrane subunit TssM	4272	133	31.1
**CPBP_00937**	type VI secretion system protein TssA	1020	46	45.1
**CPBP_00985**	type VI secretion system contractile sheath small subunit, TssB	513	22	42.9
**CPBP_00986**	type VI secretion system contractile sheath large subunit, TssC	1455	21	14.4
**CPBP_00987**	Hcp1-like superfamily protein	525	29	55.2
**CPBP_01201**	DUF4280 domain-containing protein	393	0	0.0
**Toxin-Antitoxin System 1**				
**CPBP_00210**	hypothetical protein	735	50	68.0
**CPBP_00211**	hypothetical protein	1455	131	90.0
**CPBP_00212**	hypothetical protein	474	19	40.1
**CPBP_00213**	hypothetical protein	1473	59	40.1
**CPBP_00214**	hypothetical protein	207	5	24.2
**CPBP_00215**	Colicin-E2	2043	65	31.8
**CPBP_00216**	hypothetical protein	597	19	31.8
**CPBP_00217**	DUF4157 domain-containing protein	2043	76	37.2
**CPBP_00218**	hypothetical protein	486	14	28.8
**CPBP_00219**	type IV secretion protein Rhs	10938	384	35.1
**Toxin-Antitoxin System 2**				
**CPBP_00651**	hypothetical protein	216	1	4.6
**CPBP_00652**	Colicin-E2	771	9	11.7
**CPBP_00653**	hypothetical protein	486	21	43.2
**CPBP_00654**	hypothetical protein	450	34	75.6
**CPBP_00655**	hypothetical protein	597	5	8.4
**CPBP_00656**	hypothetical protein	5016	131	26.1
**Toxin-Antitoxin System 3**				
**CPBP_00960**	MafB2 adhesin	8352	384	46.0
**CPBP_00961**	hypothetical protein	519	36	69.4
**CPBP_00962**	hypothetical protein	1893	117	61.8
**CPBP_00963**	hypothetical protein	618	10	16.2
**CPBP_00964**	serine kinase	228	26	114.0
**CPBP_00965**	serine kinase	204	8	39.2
**CPBP_00966**	RHS repeat-associated core domain-containing protein	1140	50	43.9
**CPBP_00967**	hypothetical protein	249	16	64.3
**CPBP_00968**	hypothetical protein	1476	69	46.7
**CPBP_00969**	hypothetical protein	534	25	46.8
**CPBP_00970**	hypothetical protein	411	25	60.8
**CPBP_00971**	putative zinc protease	1257	46	36.6
