# Supplementary material for: Bodo saltans (Kinetoplastida) is dependent on a novel Paracaedibacter-like endosymbiont that possesses multiple putative toxin-antitoxin systems
Source: ISME J. 2021 Jan 15;15(6):1680–94. doi: 10.1038/s41396-020-00879-6 (PMC8163844; doi:10.1038/s41396-020-00879-6)
Supplement: Supplementary file 5 — Supplementary Table 4 [file 41396_2020_879_MOESM5_ESM.pdf]

Supplementary Table 4: List of genes in *Candidatus* Bodocaedibacter vickermanii encoding for putative Type VI secretion effectors

S. No.	Gene ID	Product	T6SE	Note	Prediction by other methods
1	CPBP_00008	hypothetical protein	Yes		SignalP and SecretomeP
2	CPBP_00009	ATP-dependent RNA helicase DbpA	Yes		SecretomeP
3	CPBP_00012	peptidase	Yes		
4	CPBP_00027	CDGSH iron-sulfur domain-containing protein	Yes		SecretomeP
5	CPBP_00039	mannosyltransferase 1 family protein	Yes		
6	CPBP_00062	hypothetical protein	Yes		SignalP and SecretomeP
7	CPBP_00129	Flagellar hook-length control protein FliK	Yes		SecretomeP
8	CPBP_00131	Flagellar hook protein FlgE	Yes		SecretomeP
9	CPBP_00132	Flagellar hook protein FlgE	Yes		SignalP and SecretomeP
10	CPBP_00138	hypothetical protein	Yes		SecretomeP
11	CPBP_00143	Single-stranded DNA-binding protein	Yes		SecretomeP
12	CPBP_00151	RNA-splicing ligase RtcB	Yes		
13	CPBP_00156	phage portal protein	Yes		
14	CPBP_00163	phage major capsid protein	Yes		SecretomeP
15	CPBP_00166	hypothetical protein	Yes		SecretomeP
16	CPBP_00168	hypothetical protein	Yes		
17	CPBP_00169	hypothetical protein	Yes		
18	CPBP_00174	hypothetical protein	Yes		
19	CPBP_00184	hypothetical protein	Yes		SecretomeP
20	CPBP_00193	DsbA family protein	Yes		SignalP and SecretomeP
21	CPBP_00211	hypothetical protein	Yes	Toxin-Antitoxin system-1 (210:219)	SecretomeP
22	CPBP_00212	hypothetical protein	Yes		
23	CPBP_00213	hypothetical protein	Yes		SecretomeP
24	CPBP_00215	Colicin-E2	Yes		
25	CPBP_00217	DUF4157 domain-containing protein	Yes		
26	CPBP_00220	hypothetical protein	Yes		SecretomeP
27	CPBP_00222	Ribonuclease YobL	Yes		SignalP
28	CPBP_00244	Cellulosome-anchoring protein	Yes		SecretomeP
29	CPBP_00247	hypothetical protein	Yes		SignalP and SecretomeP
30	CPBP_00257	hypothetical protein	Yes	T6SS (258)	SignalP and SecretomeP
31	CPBP_00270	TIGR02452 family protein	Yes		SignalP and SecretomeP
32	CPBP_00289	hypothetical protein	Yes		SignalP
33	CPBP_00290	hypothetical protein	Yes		SecretomeP
34	CPBP_00297	hypothetical protein	Yes		SecretomeP
35	CPBP_00315	hypothetical protein	Yes		SecretomeP
36	CPBP_00326	filamentous hemagglutinin N-terminal domain-containing protein	Yes		

37	CPBP_00329	Murein hydrolase activator NlpD	Yes		SecretomeP
38	CPBP_00332	hypothetical protein	Yes		SignalP and SecretomeP
39	CPBP_00347	prepilin-type N-terminal cleavage/methylation domain-containing protein	Yes		SecretomeP
40	CPBP_00348	type II secretion system protein	Yes		
41	CPBP_00352	toxin-antitoxin system, YwqK family antitoxin	Yes		SecretomeP
42	CPBP_00353	type VI secretion system tip protein VgrG	Yes	T6SS	SecretomeP
43	CPBP_00354	hypothetical protein	Yes		SecretomeP
44	CPBP_00355	type VI secretion system tip protein VgrG	Yes	T6SS	
45	CPBP_00357	hypothetical protein	Yes		SignalP and SecretomeP
46	CPBP_00363	Aconitate hydratase A	Yes		
47	CPBP_00382	hypothetical protein	Yes		SignalP and SecretomeP
48	CPBP_00396	hypothetical protein	Yes		SecretomeP
49	CPBP_00413	hypothetical protein	Yes		SignalP and SecretomeP
50	CPBP_00419	DDE transposase	Yes		SecretomeP
51	CPBP_00420	Chaperone protein DnaJ	Yes		SecretomeP
52	CPBP_00430	DUF2282 domain-containing protein	Yes		SignalP and SecretomeP
53	CPBP_00432	hypothetical protein	Yes		SecretomeP
54	CPBP_00441	hypothetical protein	Yes		SignalP and SecretomeP
55	CPBP_00442	hypothetical protein	Yes		SignalP and SecretomeP
56	CPBP_00452	Dipeptidyl aminopeptidase BIII	Yes		
57	CPBP_00473	hypothetical protein	Yes		SecretomeP
58	CPBP_00484	hypothetical protein	Yes		SecretomeP
59	CPBP_00528	zinc-ribbon domain-containing protein	Yes		
60	CPBP_00535	hypothetical protein	Yes		SecretomeP
61	CPBP_00573	50S ribosomal protein L27	Yes		SecretomeP
62	CPBP_00582	NADH dehydrogenase	Yes		SecretomeP
63	CPBP_00638	flagellar hook protein FliD	Yes		
64	CPBP_00646	hypothetical protein	Yes		SignalP and SecretomeP
65	CPBP_00652	Colicin-E2	Yes	Toxin-Antitoxin	SecretomeP
66	CPBP_00653	hypothetical protein	Yes	system-2	
67	CPBP_00654	hypothetical protein	Yes	(651:656)	
68	CPBP_00663	hypothetical protein	Yes		SignalP and SecretomeP
69	CPBP_00667	hypothetical protein	Yes		SecretomeP
70	CPBP_00685	hypothetical protein	Yes		SignalP and SecretomeP
71	CPBP_00696	AraC family transcriptional regulator	Yes		SecretomeP
72	CPBP_00697	Vitamin B12-dependent ribonucleoside-diphosphate reductase	Yes		
73	CPBP_00761	hypothetical protein	Yes		
74	CPBP_00764	Murein DD-endopeptidase MepM	Yes		SecretomeP
75	CPBP_00767	Tol-Pal system protein TolB	Yes		
76	CPBP_00773	Poly(3-hydroxyalkanoate) polymerase subunit PhaC	Yes		SecretomeP
77	CPBP_00782	50S ribosomal protein L31	Yes		SecretomeP
78	CPBP_00792	2-oxoglutarate dehydrogenase E1 component	Yes		

79	CPBP_00796	hypothetical protein	Yes		SecretomeP
80	CPBP_00797	hypothetical protein	Yes		SecretomeP
81	CPBP_00798	hypothetical protein	Yes		SecretomeP
82	CPBP_00799	putative transcriptional regulatory protein	Yes		
83	CPBP_00810	Putative penicillin-binding protein PbpX	Yes		SignalP and SecretomeP
84	CPBP_00842	DUF3576 domain-containing protein	Yes		SignalP
85	CPBP_00843	Leucine--tRNA ligase	Yes		SecretomeP
86	CPBP_00854	Flagellar basal-body rod protein FlgF	Yes		SecretomeP
87	CPBP_00880	hypothetical protein	Yes		SecretomeP
88	CPBP_00900	hypothetical protein	Yes		
89	CPBP_00932	hypothetical protein	Yes	T6SS (933:937)	SecretomeP
90	CPBP_00943	Superoxide dismutase [Fe]	Yes		SecretomeP
91	CPBP_00951	Virginiamycin A acetyltransferase	Yes		
92	CPBP_00962	hypothetical protein	Yes	Toxin-Antitoxin system-3 (960:971)	
93	CPBP_00975	hypothetical protein	Yes		SignalP and SecretomeP
94	CPBP_00987	Hcp1-like superfamily protein	Yes	T6SS (985:987)	
95	CPBP_00991	Co2+/Mg2+ efflux protein ApaG	Yes		SecretomeP
96	CPBP_00998	Cysteine--tRNA ligase	Yes		
97	CPBP_01003	hypothetical protein	Yes		SecretomeP
98	CPBP_01023	hypothetical protein	Yes		SecretomeP
99	CPBP_01026	hypothetical protein	Yes		SecretomeP
100	CPBP_01049	hypothetical protein	Yes		SecretomeP
101	CPBP_01060	Exodeoxyribonuclease III	Yes		
102	CPBP_01063	hypothetical protein	Yes		SignalP and SecretomeP
103	CPBP_01064	hypothetical protein	Yes		SecretomeP
104	CPBP_01071	Penicillin-binding protein 1F	Yes		SecretomeP
105	CPBP_01134	hypothetical protein	Yes		SignalP and SecretomeP
106	CPBP_01139	putative exported protein	Yes		SecretomeP
107	CPBP_01149	hypothetical protein	Yes		SignalP
108	CPBP_01150	hypothetical protein	Yes		SecretomeP
109	CPBP_01162	fucose permease	Yes		
110	CPBP_01180	hypothetical protein	Yes		SignalP and SecretomeP
111	CPBP_01186	hypothetical protein	Yes		SecretomeP
112	CPBP_01191	hypothetical protein	Yes		SecretomeP
113	CPBP_01193	DDE transposase	Yes		SecretomeP
114	CPBP_01194	hypothetical protein	Yes		SignalP and SecretomeP
115	CPBP_01235	hypothetical protein	Yes		SecretomeP
116	CPBP_01258	outer membrane protein assembly factor BamE	Yes		SignalP and SecretomeP
117	CPBP_01259	hypothetical protein	Yes		
