# Supplementary material for: Bodo saltans (Kinetoplastida) is dependent on a novel Paracaedibacter-like endosymbiont that possesses multiple putative toxin-antitoxin systems
Source: ISME J. 2021 Jan 15;15(6):1680–94. doi: 10.1038/s41396-020-00879-6 (PMC8163844; doi:10.1038/s41396-020-00879-6)
Supplement: Supplementary file 7 — Supplementary Table 6 [file 41396_2020_879_MOESM7_ESM.pdf]

Supplementary Table 6: Annotation of polymorphic toxin systems by distant homology detection with HHsearch

**Gene**	**Module position**	**Description based on position and/or matching families**	**Matching Pfam or PDB entry suggestive of toxin or antitoxin function**	**Matching probability (%), sequence identity of HHsearch alignment**
Polymorphic Toxin System I				
CPBP_00219		Complete multidomain toxin	AHH nuclease domain (PF14412)	93, 23
CPBP_00218		Antitoxin	Gmx_para_CXXCG (PF09535); DUF1629 (PF07791); Immunity protein 43 (PF15570)	100, 14;<br>99, 15;<br>96, 16
CPBP_00217	Orphan module 1	Alternate toxin domain	Tox-HNH-HHH nuclease domain (PF15637)	100, 34
CPBP_00216		Antitoxin	SUKH superfamily 5 (PF14567)	88, 15
CPBP_00215	Orphan module 2	Alternate toxin domain	Cloacin; Colicin-like bacteriocin tRNase domain (PF03515)	99, 6
CPBP_00214		Antitoxin	Colicin/pyocin immunity protein (PF01320)	100, 16
CPBP_00213	Probable orphan module 3	Alternate toxin domain	*No strong match to any family or structure*	
CPBP_00212		Antitoxin	*No strong match to any family or structure*	
CPBP_00211	Orphan module 4	Alternate toxin domain	DUF1837 (PF08878)	81, 16
CPBP_00210		Antitoxin	Immunity protein 49 (PF15575)	100, 12

Polymorphic Toxin System II				
CPBP_00656		Complete multidomain toxin	Tox-HNH-HHH nuclease domain (PF15637)	99, 40
CPBP_00655		Antitoxin	SUKH superfamily 5 (PF14567)	91, 15
CPBP_00654	Orphan module 1	Alternate toxin domain	DNase/tRNase domain of colicin-like bacteriocin (PF12639)	100, 28
CPBP_00653		Antitoxin	SUKH superfamily 5 (PF14567)	100, 12
CPBP_00652	Orphan module 2	Alternate toxin domain	S-type pyocin (PF06958)	99, 9
CPBP_00651		Antitoxin	Colicin/pyocin immunity protein (PF01320)	100, 13
Polymorphic Toxin System III				
CPBP_00960		Presumed complete multidomain toxin	*No strong match to any family or structure*	
CPBP_00961		Presumed antitoxin	*No strong match to any family or structure*	
CPBP_00962	Possible Orphan module 1	Alternate toxin domain	*No strong match to any family or structure*	
CPBP_00963		Antitoxin	DUF3885 (PF13021)	100, 32
CPBP_00964	Not an orphan module	-	*Short, uninformative matches against a variety of Pfam and PDB entries*	
CPBP_00965		-	*Short, uninformative matches against a variety of Pfam and PDB entries*	
CPBP_00966	Possible Orphan module 2	Alternate toxin domain	Metallopeptidase toxin 5 (PF15641)	98, 12
CPBP_00967		Antitoxin	Colicin-like immunity protein (PF09204)	92, 13
CPBP_00968	Possible Orphan module 3	Alternate toxin domain	*No strong match to any family or structure*	
CPBP_00969		Antitoxin	*No strong match to any family or structure*	

CPBP_00970	Unlikely Orphan module 4	Alternate toxin domain	*No strong match to any family or structure*	
CPBP_00971		Antitoxin	Metalloprotease (PF03410)	100, 38
